# A systems approach using Diversity Outbred mice distinguishes the cardiovascular effects and genetics of circulating GDF11 from those of its homolog, myostatin

**DOI:** 10.1093/g3journal/jkab293

**Published:** 2021-09-02

**Authors:** Abigail E Starcher, Kristen Peissig, James B Stanton, Gary A Churchill, Dunpeng Cai, Joshua T Maxwell, Arthur Grider, Kim Love, Shi-You Chen, Amanda E Coleman, Emma Strauss, Robert Pazdro

**Affiliations:** 1 Department of Nutritional Sciences, University of Georgia, Athens, GA 30602, USA; 2 Department of Pathology, University of Georgia College of Veterinary Medicine, Athens, GA 30602, USA; 3 The Jackson Laboratory, Bar Harbor, ME 04609, USA; 4 Department of Physiology, University of Georgia College of Veterinary Medicine, Athens, GA 30602, USA; 5 Department of Pediatrics, Emory School of Medicine, Atlanta, GA 30322, USA; 6 K. R. Love Quantitative Consulting and Collaboration, Athens, GA 30605, USA; 7 Department of Small Animal Medicine & Surgery, University of Georgia College of Veterinary Medicine, Athens, GA 30602, USA

**Keywords:** growth differentiation factor 11, myostatin, cardiac hypertrophy, Diversity Outbred

## Abstract

Growth differentiation factor 11 (GDF11) is a member of the TGF-β protein family that has been implicated in the development of cardiac hypertrophy. While some studies have suggested that systemic GDF11 protects against cardiomyocyte enlargement and left ventricular wall thickening, there remains uncertainty about the true impact of GDF11 and whether its purported effects are actually attributable to its homolog myostatin. This study was conducted to resolve the statistical and genetic relationships among GDF11, myostatin, and cardiac hypertrophy in a mouse model of human genetics, the Diversity Outbred (DO) stock. In the DO population, serum GDF11 concentrations positively correlated with cardiomyocyte cross-sectional area, while circulating myostatin levels were negatively correlated with body weight, heart weight, and left ventricular wall thickness and mass. Genetic analyses revealed that serum GDF11 concentrations are modestly heritable (0.23) and identified a suggestive peak on murine chromosome 3 in close proximity to the gene *Hey1*, a transcriptional repressor. Bioinformatic analyses located putative binding sites for the HEY1 protein upstream of the *Gdf11* gene in the mouse and human genomes. In contrast, serum myostatin concentrations were more heritable (0.57) than GDF11 concentrations, and mapping identified a significant locus near the gene *FoxO1*, which has binding motifs within the promoter regions of human and mouse myostatin genes. Together, these findings more precisely define the independent cardiovascular effects of GDF11 and myostatin, as well as their distinct regulatory pathways. *Hey1* is a compelling candidate for the regulation of GDF11 and will be further evaluated in future studies.

## Introduction 

The heart adapts to excessive loading stresses by eccentric or concentric hypertrophy. Eccentric cardiac hypertrophy, a typical response to excessive ventricular preload, is characterized by dilatation of the affected ventricular chamber (Mihl *et al.* 2008), whereas concentric cardiac hypertrophy, a typical response to excessive ventricular afterload, is associated with increases in cardiomyocyte size and left ventricular wall thickness—features that, while initially adaptive, may ultimately contribute to the development of heart failure (Müller and Dhalla 2013). Heart failure is a major public health problem that currently affects 6.5 million Americans (Benjamin *et al.* 2019), roughly 3 million of whom will die within the next five years from related complications (Chen-Scarabelli *et al.* 2015). The cumulative prevalence of cardiac hypertrophy and heart failure is projected to increase dramatically in the coming decades, driven by a rapid population increase in those aged 65 and over, the group most profoundly affected by these conditions (Azad and Lemay 2014).

In recent years, evidence has shown that age-related cardiac hypertrophy is governed, at least in part, by systemic factors. Using heterochronic parabiosis, Loffredo and colleagues demonstrated that blood from young mice reverses signs of cardiac hypertrophy in aged animals (Loffredo *et al.* 2013), an effect seemingly mediated by the circulating protein growth differentiation factor 11 (GDF11), a member of the transforming growth factor-β (TGF-β) superfamily (Loffredo *et al.* 2013; Poggioli *et al.* 2016). Circulating GDF11 levels were found to decrease during aging, and restoring GDF11 in aged mice improved histopathological indicators of cardiac hypertrophy, recapitulating the effect of young blood (Loffredo *et al.* 2013). However, the findings of subsequent studies have called into question the true impact of GDF11 on cardiac hypertrophy (Egerman *et al.* 2015; Rodgers and Eldridge 2015; Smith *et al.* 2015; Rodgers 2016; Schafer *et al.* 2016), introducing the possibility that GDF11 has a pro-hypertrophic effect, or even no effect at all. Discrepant findings may be attributed in part to methodological limitations of many studies, with the most notable being the use of antibody-based methods that are fundamentally unable to distinguish between GDF11 and its homolog, myostatin (Egerman *et al.* 2015; Poggioli *et al.* 2016). Both proteins belong to the activin/myostatin subclass of the TGF-β superfamily, and these factors share 90% sequence identity within their signaling domains (Walker *et al.* 2016; Fan *et al.* 2017). Their disulfide-linked dimer ligands bind the same ActRIIA, ActRIIB, ALK4, and ALK5 receptors, and induce phosphorylation of SMAD2/3 transcription factors (Fan *et al.* 2017). Though myostatin has an established, anti-hypertrophic effect on muscle (Tobin and Celeste 2005; Carnac *et al.* 2007; Morissette *et al.* 2009; Rodriguez *et al.* 2014), including a direct regulatory effect on cardiomyocytes (Lee and McPherron 1999; Morissette *et al.* 2006; McKoy *et al.* 2007; Biesemann *et al.* 2014), it still remains unclear whether GDF11 has the same, or distinct, effects on cardiac hypertrophy (Smith *et al.* 2015; Fan *et al.* 2017).

This study was conducted to precisely resolve the relationships among GDF11, myostatin, and cardiac hypertrophy, while simultaneously comparing their genetic architectures to uncover any mechanisms that link them. Circulating GDF11 and myostatin levels were quantified via mass spectrometry to distinguish between these factors in the Diversity Outbred (DO) stock, a translationally relevant model of human genetic diversity, vastly expanding upon previous results gathered using inbred mice. Overall, we discovered unique relationships among GDF11, myostatin, and indicators of cardiac hypertrophy, as well as distinct loci and candidate genes behind each phenotype, and our results point to molecular pathways that will be interrogated in future studies of GDF11 and the heart.

## Materials and methods

### Animals

DO mice (*N* = 225) were purchased from The Jackson Laboratory (J: DO, JAX stock #009376) (Churchill *et al.* 2012), and arrived at the University of Georgia at about 5 weeks of age. All animals were housed under conventional conditions in the animal care facilities and received humane care in compliance with the Principles of Laboratory Animal Care formulated by the National Society for Medical Research and the Guide for the Care and Use of Laboratory Animals. The cohort contained an approximately equal number of males and females. Mice were maintained on a 12-h light-dark cycle and were given *ad libitum* access to water and standard chow (LabDiet, St. Louis, MO, USA, product 5053). Data from eight mice that died or were euthanized before the end of the study due to injuries sustained in fights or other health issues were not included in final histological analyses.

### Blood sampling and protein quantification

Blood was collected from the submandibular vein, according to protocols approved by the University of Georgia Animal Care and Use Committee. Serum was isolated and sent to the Brigham Research Assay Core at Brigham and Women’s Hospital where GDF11 and myostatin levels were quantified by LC-MS/MS. Briefly, the serum was denatured, reduced, alkylated, and subjected to pH-based fractionation via cation ion exchange SPE, then the elution fraction was digested with trypsin. The concentrated peptide mixture was eluted by liquid chromatography followed by mass spectrometric analysis. Unique proteotypic peptides from GDF11 and myostatin as well as heavy-labeled unique peptides were used for quantification. Batch effects for GDF11 and myostatin were corrected using the ComBat algorithm.

### Echocardiography

Transthoracic echocardiography was performed on DO mice at 16 weeks of age using a VisualSonics Vevo 1100 Imaging System (Toronto, Canada) with a 30-MHz probe. Mice were anesthetized with inhaled 1–2% isoflurane in oxygen and placed in a supine position on a heating platform. M-mode recordings of the left ventricle were obtained from a short-axis view at the level of the mitral valve chordal attachments to the papillary muscles. From these images, measurements from the average of 3–5 consecutive beats were used to calculate the following parameters: interventricular septal thickness at end-diastole and end-systole (IVSd and IVSs, respectively), left ventricular internal diameter at end-diastole and end-systole (LVIDd and LVIDs, respectively) and left ventricular posterior wall thickness at end-diastole and end-systole (LVPWd and LVPWs, respectively). Ejection fraction, fractional shortening, left ventricular mass, and LV volume at end-diastole and end-systole (LVVd and LVVs, respectively) were calculated from these measurements. This procedure was approved by the Institutional Animal Care and Use Committee of the University of Georgia.

### Histopathology

At 5–6 months of age, mice were euthanized, and hearts were isolated and fixed in neutral-buffered 10% formalin for 24 h at room temperature and then paraffin embedded using routine methods. Tissue was serially sectioned and stained using hematoxylin and eosin (for routine histopathologic analysis), Gordon and Sweet’s reticulin stain (for determination of cardiomyocyte cross-sectional area), and Masson’s trichrome stain (for determination of percent fibrosis). Left ventricular wall thickness, cardiomyocyte cross-sectional area, and percent fibrosis were measured using FIJI software (ImageJ).

### Genetic analyses

Tail tips were collected and sent to NEOGEN Genomics (Lincoln, NE) for DNA isolation and genotyping via the Giga Mouse Universal Genotyping Array (GigaMuga) (Morgan *et al.* 2015), on the Illumina Infinium platform. Genotypes and phenotypic data were imported into the R/qtl2 software for genetic mapping (Broman *et al.* 2019). Genotype probabilities were calculated based on the single nucleotide polymorphism (SNP) genotypes using a hidden Markov Model (Broman and Sen 2009). Mapping analysis was performed to determine the associations between genotype and phenotype and accounted for kinship using the “leave one chromosome out” method (Yang *et al.* 2014; Broman *et al.* 2019). Sex was included as a covariate in the genome scans. Statistical significance thresholds were established through permutation tests (Churchill and Doerge 1994). For significant quantitative trait locus (QTL) peaks positions, Bayesian credible intervals were calculated to identify the QTL interval. Genes with expression QTL within those intervals were then identified. Genetic mapping results are reported at a genome-wide adjusted family-wise error rate of 0.05, separately for each trait in the mapping analysis. The genome-wide adjustment is a stringent correct for testing multiple markers in the genetic mapping analysis. We did not apply a correction for mapping multiple traits. We mapped a total of 15 traits, but not all traits are independent, as some represent different normalizations of the same underlying data.

Bioinformatics was used to illuminate likely binding sites for HEY1 and FOXO1 upstream of their putative target genes. Coordinates of the transcription factors were determined in both the human (hg38) and mouse genome (mm10) through the Integrative Genomics Viewer software (version 2.8.2) (Thorvaldsdóttir *et al.* 2013). Most likely binding sites were determined by proximity to the promoter region of the gene of interest, determined by Ensembl (Howe *et al.* 2020), and by acetylation activity seen through the UCSC Genome Browser (http://genome.ucsc.edu/, last accessed Aug. 2021) using the Human Assembly Dec. 2013 (GRCh38/hg38) and Mouse Assembly December 2011 (GRCm38/mm10) versions for the human and mouse genomes, respectively.

### Statistical analyses

Mass spectrometry data were batch corrected with the Combat algorithm (Johnson *et al.* 2007) prior to analysis. Pearson correlation coefficient was performed on all anthropometric, histological, echocardiogram, and serum data (after normalizing transformations and batch corrections). Correlations were reported as significant at a *P*-value of less than 0.05. In Tables 1 and 2, we report *P*-values for the evaluation of 48 and 66 correlation statistics, respectively. The tests are not independent because we report results from separate tests for mice of each sex alongside the pooled test results. For simplicity, we report raw *P*-values without multiple testing adjustments. However, as all test results are presented, it is straightforward to evaluate significance in light of the number of test performed.

**Table 1:** Relationships among serum GDF11 and myostatin concentrations and indicators of cardiac hypertrophy in DO mice

Phenotype	GDF11 *r*, *P*-value			ln(Myostatin) *r*, *P*-value		
	Overall^*a*^	*M^b^*	*F^c^*	Overall^*a*^	*M^b^*	*F^c^*
ln(Body weight)	−0.016, 0.81	−0.18, 0.069	−0.048, 0.62	−0.34, <0.001^*^	−0.21, 0.033^*^	−0.30, 0.001^*^
ln(Heart weight)	0.042, 0.54	0.011, 0.91	−0.11, 0.26	−0.29, <0.001^*^	−0.14, 0.15	−0.21, 0.024^*^
Tibia length	−0.075, 0.28	0.042, 0.67	−0.15, 0.13	−0.046, 0.50	−0.10, 0.29	0.015, 0.88
ln(Heart weight/Body weight)	0.075, 0.27	0.19, 0.053	−0.047, 0.62	0.007, 0.91	0.052, 0.60	0.10, 0.30
ln(Heart weight/Tibia length)	0.065, 0.35	0.003, 0.98	−0.052, 0.59	−0.29, <0.001^*^	−0.13, 0.20	−0.22, 0.019^*^
Wall thickness	0.005, 0.94	0.078, 0.43	−0.20, 0.036^*^	−0.18, 0.008^*^	−0.034, 0.73	−0.094, 0.33
Cardiomyocyte cross sectional area	0.14, 0.046^*^	0.053, 0.59	0.12, 0.20	−0.11, 0.10	−0.005, 0.96	0.076, 0.43
Percent fibrosis	0.015, 0.82	−0.012, 0.90	0.054, 0.58	−0.12, 0.070	−0.20, 0.040^*^	−0.035, 0.71

In DO mice, serum GDF11 and ln(myostatin) levels were quantified by LC-MS/MS and body weight and indicators of cardiac hypertrophy were measured. Statistical parametric correlations between serum GDF11 and myostatin concentrations and other variables were determined via Pearson correlation coefficient, and *P*-values less than 0.05 were deemed to be statistically significant.

a *N* = 217.

b *N* = 106.

c *N* = 111.

*Indicates statistical significance.

**Table 2:** Relationships among serum GDF11 and myostatin concentrations and indicators of heart function in DO mice

Phenotype	GDF11 *r, P*-value			Myostatin *r*, *P*-value		
	Overall^*a*^	*M^b^*	*F^c^*	Overall^*a*^	*M^b^*	*F^c^*
ln(IVSs)	−0.23, 0.23	−0.020, 0.91	−0.23, 0.23	−0.18, 0.17	−0.23, 0.19	−0.046, 0.81
IVSd	−0.27, 0.15	0.11, 0.56	−0.27, 0.15	−0.13, 0.33	−0.30, 0.086	0.030, 0.88
LVIDs	0.046, 0.81	−0.093, 0.61	0.046, 0.81	−0.21, 0.10	0.004, 0.98	−0.33, 0.071
LVIDd	0.081, 0.67	−0.067, 0.71	0.081, 0.67	−0.23, 0.067	−0.003, 0.99	−0.31, 0.098
ln(LVPWs)	0.025, 0.90	−0.003, 0.99	0.025, 0.90	−0.085, 0.51	−0.14, 0.46	0.11, 0.57
ln(LVPWd)	−0.12, 0.53	0.052, 0.77	−0.12, 0.53	−0.091, 0.48	−0.26, 0.14	0.17, 0.38
Ejection fraction	−0.004, 0.98	0.070, 0.70	−0.004, 0.98	0.12, 0.35	−0.021, 0.91	0.28, 0.14
Fractional shortening	−0.007, 0.97	0.050, 0.78	−0.007, 0.97	0.11, 0.40	−0.029, 0.87	0.28, 0.14
LV mass	−0.15, 0.42	0.033, 0.86	−0.15, 0.42	−0.34, 0.006^*^	−0.38, 0.029^*^	−0.14, 0.46
ln(LVVs)	0.064, 0.74	−0.026, 0.88	0.064, 0.74	−0.18, 0.16	0.036, 0.84	−0.35, 0.061
LVVd	0.062, 0.75	−0.11, 0.55	0.062, 0.75	−0.24, 0.058	−0.030, 0.87	−0.31, 0.10

A total of 64 mice (34 males; 30 females) were randomly selected for echocardiography at approximately 16 weeks of age. Non-normally distributed data were adjusted by a natural log transformation and statistical correlations between serum GDF11 and myostatin concentrations and measures of heart function were determined via Pearson correlation coefficient. A *P*-value less than 0.05 was considered statistically significant. Myostatin levels were normally distributed in this subset and were therefore not transformed.

a *N* = 64.

b *N* = 34.

c *N* = 30.

*Indicates statistical significance.

IVSd, interventricular septum thickness at end-diastole; IVSs, interventricular septum thickness at end-systole; LVIDd, left ventricular internal dimension at end-diastole; LVIDs, left ventricular internal dimension at end-systole; LVPWd, left ventricular posterior wall thickness at end-diastole; LVPWs, left ventricular posterior wall thickness at end-systole; LV Mass, left ventricular mass; LVVd, left ventricular volume at end-diastole; LVVs, left ventricular volume at end-systole.

## Results and discussion

**Figure 1:**
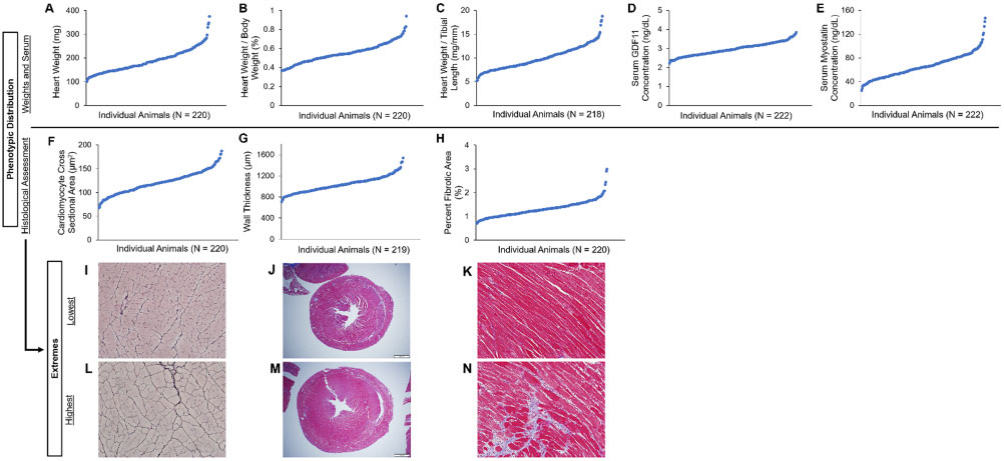
Distributions of cardiac hypertrophy markers and serum concentrations of GDF11 and myostatin in adult DO mice. The plotted distribution of heart weight (A; CI: 181, 194 mg); heart weight, as a percentage of total body weight (B; CI: 0.538, 0.566); heart weight, as a ratio to tibial length (C; CI: 9.64, 10.3); uncorrected serum GDF11 concentrations, expressed as ng/dL (D; CI: 2.92, 3.01); serum myostatin concentrations, expressed as ng/dL (E; CI: 63.2, 69.0); cardiomyocyte cross-sectional area, in µm^2^; (F; CI: 118, 124); left ventricular wall thickness, in µm (G; CI: 1018, 1059), and percent fibrotic area (H; CI: 1.27, 1.36) are displayed in the scatter plots above. Sample histology images are provided to illustrate the ranges in cardiomyocyte cross-sectional area (I, L), left ventricular wall thickness (J, M), and percent fibrotic area (K, N).

In a genetically and phenotypically diverse population of DO mice (*N* = 217; Figure 1), we discovered a significant positive correlation between serum GDF11 levels and cardiomyocyte cross-sectional area (*r* = 0.14, *P* = 0.046; Table 1). When grouped by sex, no significant correlations were found in males, but in females, a negative relationship between GDF11 and postmortem heart wall thickness (*r* = −0.20, *P* = 0.036) emerged. In the 64 DO mice (34 males; 30 females) randomly selected to undergo echocardiography, no significant correlations between GDF11 and measures of heart size or function were noted (Table 2).

In contrast to GDF11, transformed myostatin levels were negatively correlated with several measures of cardiac hypertrophy, including heart weight (natural log transformed; *r* = −0.29, *P* < 0.001), heart weight standardized to tibial length (natural log transformed; *r* = −0.29, *P* < 0.001), and left ventricular heart wall thickness (*r* = −0.18, *P* = 0.008; Table 1). We found that myostatin levels tended to be higher in females [*t*(215) = 3.72, *P* < 0.001], and similarly, heart wall thickness tended to be lower in females [*t*(217) = −8.65, *P* < 0.001; Table 1]. In addition, we found a negative correlation between myostatin and total body weight (natural log transformed; *r* = −0.34, *P* < 0.001), a trend that aligns with past evidence that myostatin negatively regulates body mass (Lee and McPherron 1999; Joulia-Ekaza and Cabello 2006; Lee 2010; Mendias *et al.* 2015). When separated by sex, males showed a negative correlation between myostatin and total body weight (*r* = −0.21, *P* = 0.033), and percent fibrotic area (*r* = −0.20, *P* = 0.040). Females showed a negative relationship between myostatin and total body weight (natural log transformed; *P* = −0.30, *P* = 0.001), heart weight (natural log transformed; *r* = −0.21, *P* = 0.024), and the ratio of heart weight to tibia length (natural log transformed; *r* = −0.22, *P* = 0.019; Table 1). In the DO subset that underwent echocardiography, a significant negative correlation emerged between untransformed myostatin levels and left ventricular mass (LVM; *r* = −0.34, *P* = 0.006). When separated by sex, the only significant correlation appeared in males: a negative correlation between serum myostatin and LVM (*r* = −0.38, *P* = 0.029; Table 2). Although these data align with the findings of previous studies establishing the anti-hypertrophic effects of myostatin (Morissette *et al.* 2009; Rodgers *et al.* 2009), other rodent studies have reported no effect of myostatin on adult and aged heart mass (Cohn *et al.* 2007), suggesting that the role of myostatin in the heart is complex and may be context-dependent.

These findings provide novel insight into the distinct relationships between GDF11, myostatin, and the heart, and most surprisingly do not support a broadly anti-hypertrophic effect for GDF11. That said, it is essential to put these data into proper context. First, this study assessed DO mice as adult animals aged 5–6 months. As such, the data highlight the fundamental relationships between circulating factors and the heart at a single time point (*i.e.*, adulthood) and may serve as the foundation for future aging studies. Second, LC-MS/MS was used to quantify serum GDF11 and myostatin levels. This technique is highly specific and more sensitive for distinguishing between GDF11 and myostatin than antibody-based methods (Semba *et al.* 2019; Camparini *et al.* 2020), yet it provides only one aspect of a more complex system. For example, these proteins may circulate freely in the active form or may be bound to inhibitor proteins, such as GASP-1 and GASP-2 (Lee and Lee 2013), which render them inactive. The methods used in this study measure total circulating concentrations of these proteins, but cannot distinguish between their active and inactive forms. It is possible that the free form of GDF11 has a unique, and stronger, relationship with the heart.

An additional focus of this study involved genetic analyses. We calculated heritabilities for serum GDF11 and myostatin concentrations, which revealed a modest heritability for GDF11 levels (0.23) and a moderate heritability for myostatin levels (0.57). The GDF11 heritability estimation is lower than the estimate (0.75) previously reported by our group, which could be explained in part by the fact that the prior study used an ELISA test that likely failed to fully discriminate between GDF11 and myostatin, resulting in a higher heritability estimate reflecting that of myostatin (Zhou *et al.* 2016).

**Figure 2:**
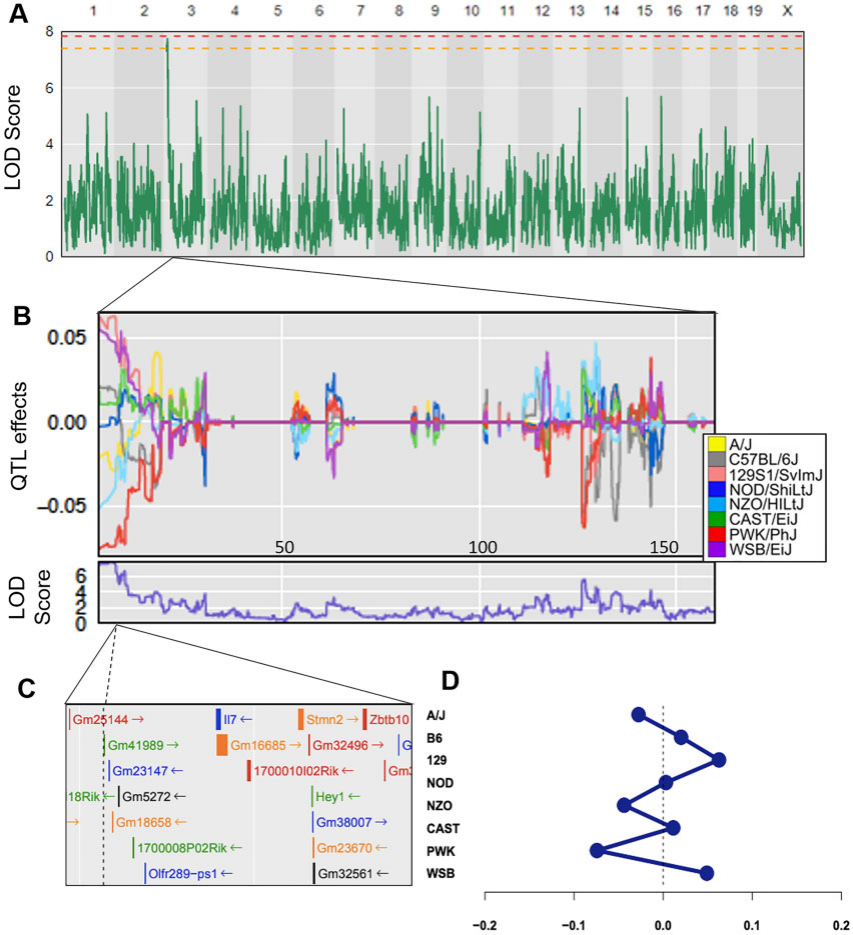
Quantitative trait locus mapping of serum GDF11, adjusted for sex, batch, and kinship. Horizontal lines represent permutation testing significant threshold (orange line at *P*-value = 0.1, red line at *P*-value = 0.05). (A) The QTL model for GDF11 revealed a suggestive peak (*P* < 0.1) at Bayesian credible interval 3.039589–9.983782 Mbp, with (B, D) additive allele effects from the contributing mouse strains. (C) Genes located near the causative SNP include *Hey1*. (D) Allele effects at the chromosome 3 peak SNP.

High-precision gene mapping was then performed using R/qtl2 software (Broman *et al.* 2019), and no significant peaks were found for phenotypes related to heart size or histology in normal adulthood. Yet it should be noted that a prior DO study identified two QTL associated with heart size in the DO stock, one reaching significance (*P* ≤ 0.05; chromosome 15 at 72.47 Mb) and the other suggestive (*P* ≤ 0.1; chromosome 10 at 120 Mb) (Shorter *et al.* 2018). Neither locus overlapped with any QTL found in this study, and the lack of heart weight QTL in our study can be attributed to the difference in power (*N* = 217 *vs N* = 547). Meanwhile, mapping serum GDF11 levels revealed a suggestive peak (*P* < 0.1) on murine chromosome 3 within the Bayesian credible interval 3.039589–9.983782 Mbp (Figure 2, A and B). The peak was located in close proximity to the protein-coding gene *Hes Related Family BHLH Transcription Factor with YRPW Motif 1* (*Hey1*; Figure 2, C and D*)*, a member of the hairy and enhancer of split-related (HESR) family of basic helix-loop-helix (bHLH) transcriptional repressors (Weber *et al.* 2015). Proteins in the HESR family repress target genes via epigenetic modification, mediated by Hdac recruitment and resulting in histone deacetylation (Weber *et al.* 2015). These proteins have been previously linked to cardiovascular development (Fischer *et al.* 2002; Rutenberg *et al.* 2006; Fischer and Gessler 2007; Weber *et al.* 2015), with high expression of Hey bHLH transcription factors, such as HEY1 and HEY2, in atrial and ventricular cardiomyocytes as well as in the endocardium (Weber *et al.* 2015). HEY1 in particular promotes heart development by participating in an important signaling cascade for the differentiation of nonchamber atrioventricular canal and inner curvature regions of the heart (Rutenberg *et al.* 2006; Fischer *et al.* 2007), and regulates expression of other transcription factors involved in cardiac development *in vitro* (Weber *et al.* 2015). In the adult mouse, members of the Hey family have shown an antihypertrophic effect on the heart (Xiang *et al.* 2006). While these findings indirectly support *Hey1* as a candidate gene behind circulating GDF11 levels, we also cannot exclude other plausible genes within the same locus, such as *Stathmin-2* (*Stmn2*). *Stmn2* is another reasonable candidate as it is expressed in both murine and human heart tissue throughout development and into adulthood (Asp *et al.* 2019; Bult *et al.* 2019; Smith *et al.* 2019).

**Figure 3:**
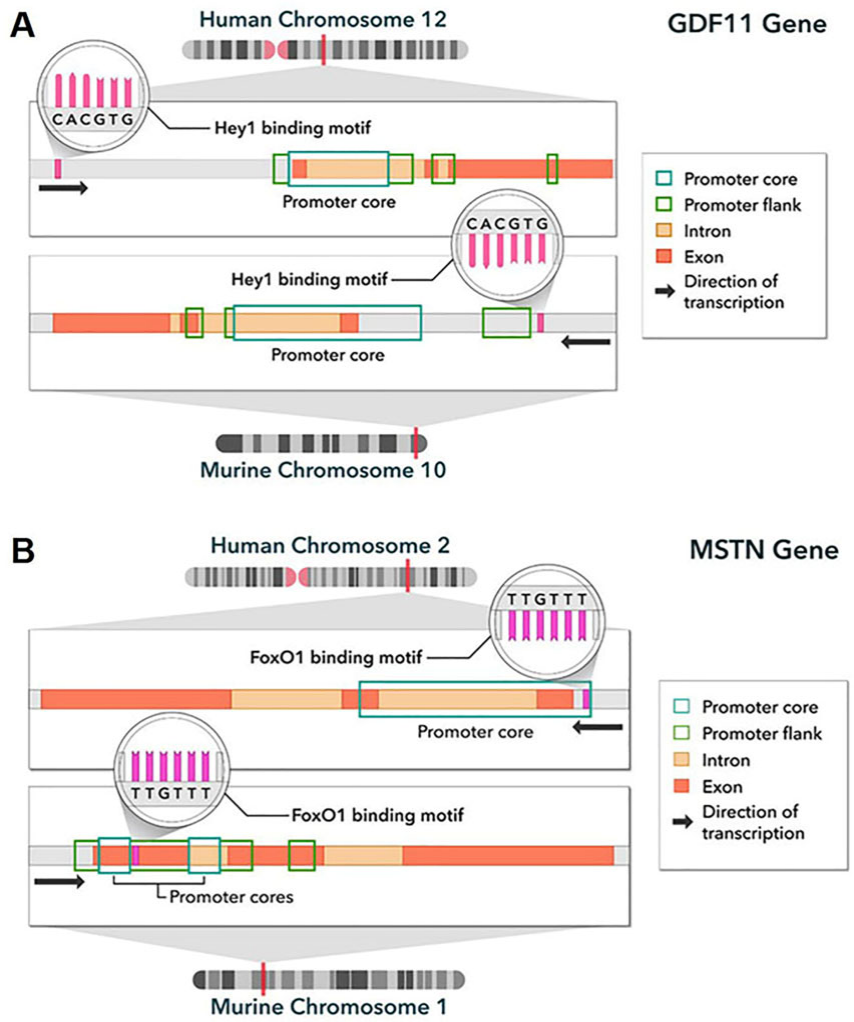
A model for transcriptional regulation of the *GDF11* and *myostatin* (*MSTN*) genes. (A) Bioinformatics revealed several HEY1 binding sequences (5′-CACGTG-3′) upstream of the *Gdf11/GDF11* gene in both humans and mice; the most likely site per species was selected and shown in proximity to the promoter region. (B) Binding sequences of the transcription factor FOXO1 were determined upstream of *Mstn/MSTN* gene in the human and mouse genomes. The most likely binding motif lies within the promoter regions in both species.

*In silico* analyses were used to test whether HEY1 has binding sites proximal to the *Gdf11* gene. HEY1 preferentially binds to the canonical E box sequence 5′-CACGTG-3′ in both the murine and human genomes (Nakagawa *et al.* 2000; Sun *et al.* 2001; Fischer and Gessler 2007); several HEY1 binding motifs were observed upstream of *Gdf11/GDF11* in both species (Thorvaldsdóttir *et al.* 2013). The binding motif on mouse chromosome 10 nearest to *Gdf11* is located at Chr10:128,898,596–128,898,601, only 1100 base pairs away from the flanking promoter region and 5196 base pairs from the gene itself (Figure 3). In the human genome, the nearest HEY1 binding site to the *GDF11* gene lies on human chromosome 12 (Chr12:55,729,183–55,729,189), located 13,933 base pairs upstream from the *GDF11* gene and 12,613 base pairs from the flanking promoter region (Figure 3). Further analysis (human genome hg38 assembly) revealed that this particular HEY1 binding sequence lies within highly active histone H3 lysine 27 acetylation (H3K27Ac) and trimethylation of histone H3 lysine 4 (H3K4Me3) regions, which are epigenetic marks strongly correlated with active transcription (Soutoglou *et al.* 2000; Kent *et al.* 2002). Furthermore, chromatin immunoprecipitation sequencing (ChIP-seq) data available through NCBI Genome Data Viewer (Rangwala *et al.* 2021) demonstrated evidence of HEY1 binding activity within the genomic region Chr12:56,122,620–56,123,650 (GRCh37; in HepG2 cells), which contained our predicted HEY1 binding site—following its conversion to the GRCh37 build (Chr12:56,122,967–56,122,973; data not shown). It should be noted that this HEY1 binding site may regulate a neighboring gene, such as CD63 (Chr12:55,725,323–55,729,707). Though the binding sites for HEY1 were located outside of the *Gdf11/GDF11* promoter regions in both humans and mice, their proximities to the gene, as well as the acetylation surrounding the motifs—particularly in human DNA—suggest that *Hey1* is a plausible candidate gene in the regulation of GDF11 via transcriptional control. We posit that the HEY1 binding site is still located within the upstream regulatory region, especially for mouse *Gdf11*, and that HEY1 inhibits co-activators from binding to enhancers in the distal regulatory regions to modulate *Gdf11* transcription. Additional research is now needed to experimentally validate this model, especially in the context of past studies. For instance, ChIP has been used to define the DNA-binding activity of HEY proteins, and their effects on gene expression (Heisig *et al.* 2012), yet the current study underscores the need for additional research that specifically focuses on the impact on *Gdf11/GDF11* expression. Further, most research supports HEY1 as a transcriptional repressor, but the related hair and enhancer of split-1 (HES1) transcription factor has documented activator activity (Ju *et al.* 2004), so the anticipated repressor role of HEY1 in this model should also be confirmed.

It must be noted that the QTL peak associated with serum GDF11 levels fell short of the *P* < 0.05 significance threshold. Since the peak was discovered using *N* = 217 DO mice, we anticipate that greater mouse numbers would have resulted in a greater level of significance (Gatti *et al.* 2014). Yet with a *P* < 0.10, the peak is considered highly suggestive and similar to peaks that have been reported in other DO mapping studies (Logan *et al.* 2013; Shorter *et al.* 2018; Schlamp *et al.* 2019), so it should not be discounted. Overall, the QTL peak underlying serum GDF11 concentrations is highly promising, and our collective confidence in the peak will be strengthened by additional exploration and validation in future genetics studies.

**Figure 4:**
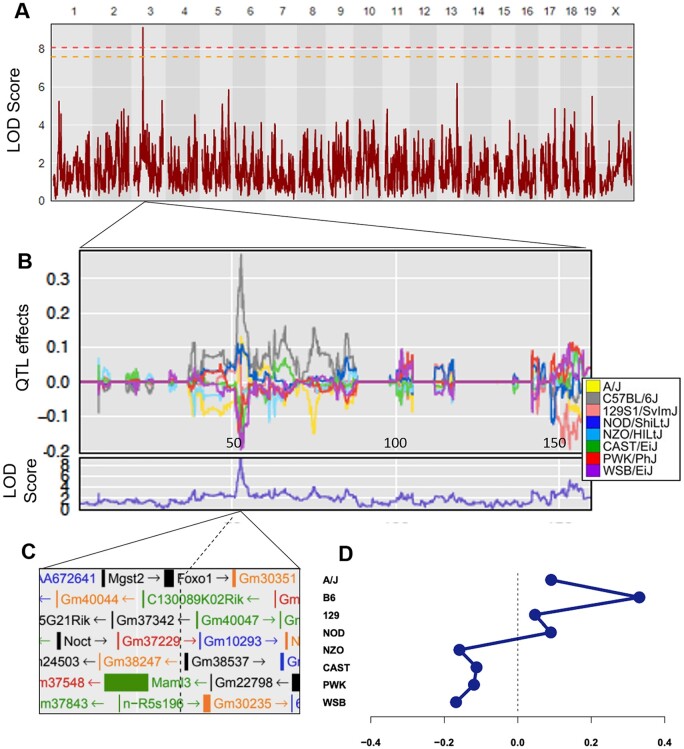
Quantitative trait locus mapping of serum myostatin, adjusted for sex, batch, and kinship. Horizontal lines represent permutation testing significant threshold (orange line at *P*-value = 0.1, red line at *P*-value = 0.05). (A) The QTL model for myostatin revealed a significant peak (*P* < 0.05) at the Bayesian credible interval of 52.26269–52.71985 Mbp, with (B, D) additive allele effects from DO parent strains plotted. (C) Genes located near the associated SNP include *FoxO1*. (D) Allele effects at the chromosome 3 peak SNP.

In parallel, we conducted genetic mapping of serum myostatin levels and discovered a significant locus (*P* < 0.05) on murine chromosome 3 within the Bayesian credible interval of 52.26269–52.71985 Mbp (Figure 4, A and B). The peak is located in close proximity to protein-coding gene *Forkhead Box O1* (*FoxO1*; Figure 4, C and D). FOXO1, along with several other Forkhead proteins, plays an essential role in cardiac development (Hosaka *et al.* 2004; Ronnebaum and Patterson 2010) and appears to be equally vital in maintaining the function of the adult heart (Ni *et al.* 2007; Ronnebaum and Patterson 2010). Multiple studies have shown that FOXO1 increases myostatin expression in myotubes (Allen and Unterman 2007; Morissette *et al.* 2009; Beharry *et al.* 2014; Xu *et al.* 2017), though one study of trout myotubes found no effect of FOXO1 on myostatin expression (Seiliez *et al.* 2011). We identified the FOXO1 binding sequence 5′-TTGTTT-3′ sites on murine chromosome 1 (Thorvaldsdóttir *et al.* 2013; Auguste *et al.* 2018); the most likely site fell within the myostatin (*Mstn)* gene itself and within the flanking promoter region (Figure 3B). This particular location (Chr1:53,062,323–53,062,328) showed moderate acetylation activity when examined using the UCSC Genome Browser (mm10 assembly), increasing the likelihood that it serves as a site for FOXO1 binding (Soutoglou *et al.* 2000; Kent *et al.* 2002). In the human genome, we searched chromosome 2 near the *MSTN* gene for same motif, TTGTTT, since the Forkhead protein is highly conserved across species (Allen and Unterman 2007), and found that it also fell within the gene’s promoter region (Kent *et al.* 2002; Figure 3B). These findings support a role for FOXO1 in the regulation of myostatin expression and suggest that genetic variants in or near the *FoxO1* gene govern circulating myostatin levels.

In summary, the findings of this study underscore a relatively weak, inconsistent relationship between total serum GDF11 levels and cardiac hypertrophy in a genetically diverse population of adult mice and support a stronger, consistent anti-hypertrophic role for its homolog, myostatin. To our knowledge, this study is the first to identify a candidate genetic regulator of serum GDF11 concentrations in adults. That gene, *Hey1*, encodes a transcriptional repressor with putative binding sites located in close proximity to the *Gdf11/GDF11* gene in the mouse and human genomes. HEY1 is part of the Notch pathway (Niessen and Karsan 2008), a signaling cascade that mediates the proliferation and differentiation of cardiomyocytes as well as remodeling the developed heart under stress (Niessen and Karsan 2008; MacGrogan *et al.* 2018). These results form the necessary foundation for future studies, which will further interrogate *Hey1* as a regulator of GDF11 and cardiovascular disease, and lead to a better understanding of the cardiovascular impact of GDF11 in older adults.

## Data availability

The DO stock is available through The Jackson Laboratory (Bar Harbor, ME; https://www.jax.org/strain/009376, last accessed Aug. 2021). The Reagent Table can be found in the Supplementary information on FigShare, as can the raw, uncorrected phenotypic data (Supplementary Table S1), the GeneSeek data containing the genotypes from each mouse (Supplementary File S1), genotype probabilities (Supplementary File S2), and the script used (Supplementary File S3). The phenotype QTL viewers are available at https://churchilllab.jax.org/qtlviewer/pazdrodoheart (last accessed Aug. 2021). Marker information containing the genetic map (cM) and physical map (Mbp) for the GigaMUGA, the eight founder strain genotypes, the GigaMUGA founder genetic maps (cM) and physical maps (Mbp) for each chromosome (Supplementary File S4), and the genetic mapping reports (Supplementary File S5) can also be found on figshare: https://doi.org/10.25387/g3.14248718.
